# Zonular Cataract and Dental Malformations

**Published:** 1886-12

**Authors:** 


### ZONULAR CATARACT AND DENTAL MALFORMATIONS.

The October number (1886) of the *Ophthalmic Review* contains an article on "Zonular Cataract and Dental Malformations," by John B. Storey, M.B., F.R.C.S.I., in which he calls attention to the frequency with which dental deformities occur in this form of cataract. He quotes the views of Professor Horner and Professor Arlt as to the causes which produce these defects in the lens and the teeth. It is stated that Professor Horner believed that the connection between zonular cataract and the peculiar dental malformations which so frequently accompany it, is due to the influence of infantile rickets, and that Professor Arlt is of the opinion that it was due to infantile convulsions. Out of sixty-five cases of zonular cataract mentioned by Horner and Arlt, forty-eight had a history of infantile convulsions, and in Horner's thirty-six cases, twenty-five had dental defects, sixteen had malformed crania, and four had imperfect mental development.

Mr. Storey also reports nine cases, in corroboration of Horner's observations, six of them with marked dental defects. In describing the malformations of the teeth he says "the dental deformity must not be confounded with that ascribed by Mr. Hutchinson to congenital syphilis. It is, on the contrary, precisely similar to what Hutchinson has attributed to the use of mercury in infancy, * * * which, by setting up stomatitis, produces the dental abnormality."

It is extremely difficult to distinguish between the characteristic features of dental malformations ascribed by Hutchinson to the effects of inherited syphilis and those due to the effects of the prolonged administration of mercury in early infancy.

The semi-lunar notch found in the cutting edges of the permanent upper central incisors, "the test teeth," has been considered as diagnostic of inherited syphilis, but inasmuch as it is unusual to find such cases in which there is not also the history of mercurial impression, it requires strong evidence to enable one to assert that such defects, in the development of these teeth, can be the result of no other cause than syphilis.

The teeth which are most often defective in development are the first permanent molars and the six anterior teeth, viz.: the incisors and cuspids. Calcification begins in these teeth during the first two years of life, or, to be more precise, in the molars, at birth, in the incisors during the first year, and in the cuspids during the second, and this is the period when the impress of disease is most marked upon the developing teeth.

Syphilis seems to have a special predilection for the tegumentary system, and often acts with great virulence upon these tissues. As the teeth are a part of this system, we can the more readily understand how nutrition of the gums of these organs would be for a considerable time perverted, and result in defective development.

The too common use of large doses of mercury in certain infantile disorders, the long-continued administration of the drug which is necessarily practiced in the treatment of syphilis, lowers the vital forces and produces a depressing effect upon the nutritive functions. Cases could be cited of results from the administration of this drug and other influences in which syphilis undoubtedly did not exist, that would make it impossible from the teeth alone to determine that the results were not due to the syphilitic virus.

On the other hand, the malformations of the teeth described by Arlt as due to infantile convulsions, by Horner to infantile rickets, and Hutchinson to the effects of mercury, cannot in many instances be distinguished from those due to the influences of the eruptive fevers, typhoid fever, or in fact any disease sufficiently severe in its character to markedly disturb the nutritive processes.

Careful observation will bear out the statement that any influence, no matter what, which depresses or arrests the process of nutrition during the calcification of the teeth, leaves upon them the indelible evidence of defective development, and the more prolonged and severe is this depression of funcon the more marked are the defects: while a correct knowledge of the periods at which calcification of the various permanent teeth begins and terminates, furnishes data from which a very reliable estimate may be made as to the time—at least to within a few months—when the disturbance to nutrition occurred.

